# Prediction of cervical cancer incidence in England, UK, up to 2040, under four scenarios: a modelling study

**DOI:** 10.1016/S2468-2667(17)30222-0

**Published:** 2017-12-19

**Authors:** Alejandra Castanon, Rebecca Landy, Francesca Pesola, Peter Windridge, Peter Sasieni

**Affiliations:** a Centre for Cancer Prevention, Wolfson Institute of Preventive Medicine, Queen Mary University of London, London, UK; b Bermondsey Wing, Guy's and St Thomas', Kings College London, London, UK

## Abstract

**Background:**

In the next 25 years, the epidemiology of cervical cancer in England, UK, will change: human papillomavirus (HPV) screening will be the primary test for cervical cancer. Additionally, the proportion of women screened regularly is decreasing and women who received the HPV vaccine are due to attend screening for the first time. Therefore, we aimed to estimate how vaccination against HPV, changes to the screening test, and falling screening coverage will affect cervical cancer incidence in England up to 2040.

**Methods:**

We did a data modelling study that combined results from population modelling of incidence trends, observable data from the individual level with use of a generalised linear model, and microsimulation of unobservable disease states. We estimated age-specific absolute risks of cervical cancer in the absence of screening (derived from individual level data). We used an age period cohort model to estimate birth cohort effects. We multiplied the absolute risks by the age cohort effects to provide absolute risks of cervical cancer for unscreened women in different birth cohorts. We obtained relative risks (RRs) of cervical cancer by screening history (never screened, regularly screened, or lapsed attender) using data from a population-based case-control study for unvaccinated women, and using a microsimulation model for vaccinated women. RRs of primary HPV screening were relative to cytology. We used the proportion of women in each 5-year age group (25–29 years to 75–79 years) and 5-year period (2016–20 to 2036–40) who have a combination of screening and vaccination history, and weighted to estimate the population incidence. The primary outcome was the number of cases and rates per 100 000 women under four scenarios: no changes to current screening coverage or vaccine uptake and HPV primary testing from 2019 (status quo), changing the year in which HPV primary testing is introduced, introduction of the nine-valent vaccine, and changes to cervical screening coverage.

**Findings:**

The status quo scenario estimated that the peak age of cancer diagnosis will shift from the ages of 25–29 years in 2011–15 to 55–59 years in 2036–40. Unvaccinated women born between 1975 and 1990 were predicted to have a relatively high risk of cervical cancer throughout their lives. Introduction of primary HPV screening from 2019 could reduce age-standardised rates of cervical cancer at ages 25–64 years by 19%, from 15·1 in 2016 to 12·2 per 100 000 women as soon as 2028. Vaccination against HPV types 16 and 18 (HPV 16/18) could see cervical cancer rates in women aged 25–29 years decrease by 55% (from 20·9 in 2011–15 to 9·5 per 100 000 women by 2036–40), and introduction of nine-valent vaccination from 2019 compared with continuing vaccination against HPV 16/18 will reduce rates by a further 36% (from 9·5 to 6·1 per 100 000 women) by 2036–40. Women born before 1991 will not benefit directly from vaccination; therefore, despite vaccination and primary HPV screening with current screening coverage, European age-standardised rates of cervical cancer at ages 25–79 years will decrease by only 10% (from 12·8 in 2011–15 to 11·5 per 100 000 women in 2036–40). If screening coverage fell to 50%, European age-standardised rates could increase by 27% (from 12·8 to 16·3 per 100 000 by 2036–40).

**Interpretation:**

Going forward, focus should be placed on scenarios that offer less intensive screening for vaccinated women and more on increasing coverage and incorporation of new technologies to enhance current cervical screening among unvaccinated women.

**Funding:**

Jo's Cervical Cancer Trust and Cancer Research UK.

## Introduction

The epidemiology of cervical cancer in high-income countries is changing. In England, UK, women vaccinated against human papillomavirus (HPV) in 2008 at age 17 years have been invited to screening for the first time in 2016–17. Furthermore, the cervical screening programme is preparing for the introduction of HPV testing as the primary screening test.^1^ So, not only will cohorts of women entering the screening programme have a lower risk of cervical cancer, but the sensitivity of the screening test to precancer will be increased. Additionally, the European Commission has in April, 2016, granted marketing authorisation for two-dose Gardasil nine vaccination (ie, the nine-valent vaccine), which protects against HPV types that cause about 90% of cervical cancers compared with the current bivalent and quadrivalent vaccines that protect against about 70% of cervical cancers.^2^

**Research in context**

**Evidence before this study**

We searched PubMed with the search terms “cancer screening” AND “projections” OR “cancer prevention” AND “projections” to identify studies with a similar study design, which would provide evidence for time trends that took into account cancer prevention activities. We did not use date or language restrictions. We noted that age period cohort models have been used to estimate the effect of screening by assuming that period effects reflect the impact of screening. However, such models cannot explore the effect of decreasing screening coverage. Dynamic transmission models consider the effects of human papillomavirus (HPV) prevalence and transmission, cervical screening, and HPV vaccination on cervical cancer incidence, and are almost always used to produce relative measures of effect. We are not aware of any dynamic transition models that allow for underlying changes in risk on the basis of cohort effect.

**Added value of this study**

Our study combined an age period cohort model (underlying rates of disease) with a dynamic model to estimate the reduction in risk of cervical cancer following screening by attendance status and vaccination status. It not only allowed for the estimation of absolute changes to incidence of cervical cancer, but also allowed us to vary screening coverage and vaccine uptake to estimate the effect on absolute rates.

In combining the three levels of modelling, this study is the first to explore the effect of introducing HPV testing, changes in screening coverage, and vaccine uptake on cervical cancer incidences over the next 25 years. The study provides the absolute impact these changes will have at a population level in England, UK. In particular, it highlights the need for continued innovation within the programme to ensure cervical cancer rates decrease. We project that given current coverage and vaccine uptake, and accounting for the introduction of HPV primary screening, rates of cervical cancer will see only a modest decrease in the next 25 years.

**Implications of all the available evidence**

Evidence presented in this study suggests the cervical screening programme will need to adapt swiftly to the changing epidemiology of cervical cancer. Going forward, focus should be placed on scenarios that offer less intensive screening for vaccinated women and more on increasing coverage and incorporating new approaches to enhance current cervical screening in unvaccinated women.

Organised cervical screening was introduced in England in 1988 to women aged 20–64 years. Since 2003, cytology-based screening is offered once every 3 years to women aged 25–49 years and once every 5 years to women aged 50–64 years. HPV bivalent vaccination was introduced in 2008 as a school-based programme to girls aged 12–13 years, although a catch-up cohort of women aged 14–18 years were also offered the vaccine.

In the next 25 years, women aged 50 years and older will not benefit from prophylactic vaccination, and with screening ceasing by age 64 years for women with negative results, one might expect the burden of cervical cancer to once again move towards older age groups. Furthermore, population projections for the UK estimate a substantial increase in the number of women older than 60 years: a 29% increase (from 9·4 million in 2012 to 12·1 million in 2037) in the number of women aged 60–74 years and a 90% increase in those older than 75 years (from 5 million to 9·5 million).^3^

Because the aim of the cervical screening programme is to prevent the development of cancer by identifying and treating precancerous disease, age-specific incidence estimates (rates and numbers of cases) of cervical cancer for the next 25 years will help policy makers and local commissioning groups to adequately determine where the demand on preventive services will be.

In this study, we aimed to estimate the age-specific incidence of cervical cancer in England over the next 25 years from 2015 to 2040 in four policy scenarios: no changes to current screening coverage or vaccine uptake and HPV primary testing from 2019 (ie, the status quo), changing the year in which HPV primary testing is introduced, introduction of the nine-valent vaccine, and changes to cervical screening coverage.

## Methods

### Model design

We did a data modelling study that combined results from three levels: population modelling of incidence trends (level 1), observable data from the individual level with use of a generalised linear model (level 2), and unobservable disease states modelled through a microsimulation (level 3). We chose this approach because the microsimulation model focuses on a single cohort of women progressing from age 12 years to 80 years. Hence, we needed age-specific cohort effects to model rates in the future. Additionally, where possible, we used individual level data because it is more accurate and requires fewer assumptions than microsimulation data. The appendix (p 1) summarises how the different components of the model were brought together. Our model software is available from the corresponding author, so that those interested can consider other scenarios.

We started by estimating the risk of cervical cancer in the absence of screening on the basis of women's individual screening histories. We used data from cases with cervical cancer diagnosed between April, 2007, and March, 2012 (and women with no history of cervical cancer matched on age and area of residence as the control), from the audit of invasive cervical cancers,^4^ a case-control study nested in a cohort that included more than 90% of cervical cancers diagnosed in England along with their full screening histories. This study design allowed us to obtain absolute risks by fitting a generalised linear model using the binomial family and log link function for each age group and staging of cervical cancer (stages 1A, 1B, and 2+), weighted so the number of cases in the audit diagnosed in each year and age group matched the number of cases recorded in the Office for National Statistics (ONS) cancer registration statistics (2011–15).^5^ These estimates were multiplied by five to estimate the 5-year risk. This first step provided absolute risks per 100 000 women by age for 2011–15 in the absence of screening and vaccination (appendix p 1).

To allow the underlying cancer incidence to vary over the next 25 years in the absence of vaccination and screening, we used a modified age period cohort model to fit cancer incidence data from 1971 to 2013 (cervical cancer incidence data were provided by the National Cancer Registration and Analysis Service [request ODR_2014_335]), and extrapolated it to 2040. The model includes a period effect, to capture the effect of screening, which was set to zero for years before the introduction of organised screening in England in 1988. To estimate future incidences in the absence of screening (ie, a counterfactual baseline), we used the combined age and cohort effects only. We used these baseline rates to calculate age-specific cohort effects relative to the 2011–15 cohort. Further details can be found in the appendix (pp 2, 3). In the second step, this model was used to obtain the risk for each period given age relative to 2011–15 in a population not offered screening or vaccination (appendix p 1). Although the age cohort model could be used to provide estimates of the absolute risks of cervical cancer among unscreened women, we chose to use risks estimated from individual level data (from the audit), because stronger assumptions are required in the age cohort model. The absolute risks from the first step were multiplied by the cohort effects of the second step to provide absolute risks of cervical cancer for unscreened women for each 5-year period from 2016–20 to 2036–40.

To allow for the effect of screening in unvaccinated women, we used relative risks of being diagnosed with cervical cancer by screening history calculated from the audit of invasive cervical cancers^4^ (appendix p 1). Hence, the relative effect of screening is dependent on age and screening history, but does not depend on the underlying risk of the birth cohort. Screening histories were categorised as never screened (either no tests or tests only more than 15 years earlier), regularly screened (screened at least once every 3·5-year [5·5 years after age 50 years] period in the previous 15 years), and lapsed attender (screened within a 15-year interval but not often enough to be defined as regularly screened). We used data from the cervical screening programme statistics (2014–15)^6^ to establish the proportion of women by age group in each of the screening history categories. We consider this to be the current screening coverage by age group in England and consider current incidence to be the observed average rate reported between 2011 and 2015 (appendix p 10). Women are not offered cervical screening beyond age 65 years in England. We estimated the risk of cervical cancer as a function of age (65–69 years, 70–74 years, and 75–79 years) in women regularly and irregularly screened at age 50–64 years relative to that in those not screened in the same age group. The risk of cancer at age 65–79 years is thereby determined by the screening history between ages 50 and 64 years.

Unvaccinated women are screened with use of cytology until HPV primary screening is implemented. We have taken the relative effectiveness of primary HPV screening compared with cytology screening from previously published research (appendix p 1), which concluded that in addition to the cervical cancer already prevented by cytology-based screening a further 24% of currently observed cases can be prevented each year once HPV screening has been fully introduced.^7^ More details are presented in the appendix (p 4).

The introduction of HPV primary screening into the programme was made under four assumptions. Firstly, in 2019, the test will be introduced to all laboratories in England at the same time. Secondly, HPV screening will not prevent any additional cancers within 12 months of the test, and the first women to benefit are those who test positive for HPV but negative with use of cytology who will be recalled for repeat testing 12 months later. Thirdly, the full effect of switching to HPV primary screening will be observed (in terms of cancer prevention) at the next screening round (ie, once every 3 years for those aged 25–49 years and once every 5 years for those aged 50–64 years). In any given year, 33% of women younger than 50 years will be invited for screening (20% of those aged 50–64 years); therefore, the full effect of the primary HPV screening roll-out will not be seen for 6 years in women aged 25–29 years and for 10 years in those aged 50–64 years (appendix p 8). Lastly, the introduction of primary HPV screening will affect women 65 years and older in the cohort who received HPV testing at age 60–64 years. No woman aged 65–69 years will be tested with HPV before 2021, whereas from 2026 onwards all women aged 65–69 years who were screened at age 60–64 years would have benefited from an HPV test.

The effect of screening in vaccinated women was estimated from a microsimulation model^8^ (appendix pp 4–7). Vaccinated women in the model are offered screening with use of HPV testing with a 6-year interval. The effect of vaccination was estimated using HPV 16/18 vaccination (ie, the bivalent or quadrivalent vaccine) and nine-valent vaccination. Vaccination with the 16/18 vaccine was assumed to prevent all HPV 16 and 18 infections as well as 15% of non-HPV 16 and 18 infections,^9^ corresponding to 74·5% of cervical cancers, whereas the nine-valent vaccine was assumed to prevent 90% of cervical cancers.^10^ The 16/18 vaccine was introduced in England and in the model in 2008–09 to girls aged 12–13 years (born from Sept 1, 1995, to Aug 31, 1996) and to a catch-up cohort aged 14–18 years (born from Sept 1, 1990, to Aug 31, 1995). Coverage among girls aged 12–13 years was 86%.^11^ The assumed effect of vaccination on the catch-up cohort is summarised in the appendix (p 8).

Changes to vaccine uptake at age 12 years cannot affect women aged 25–29 years until 2031. Before 2031, women were assumed to have a 16/18 vaccine uptake of 86% throughout the model. For women aged 30–34 years, vaccine uptake was allowed to vary from 2036. No changes in vaccine uptake were allowed for women aged 35–49 years, and no women older than 49 years were protected by vaccination by 2040.

To calculate the incidence in the population as a whole (appendix p 1), we used the proportion of women in each age category and interval who have a specific combination of screening and vaccination history, and apply these as weights to the corresponding rates of cervical cancer to obtain a weighted average. To calculate absolute numbers of cancers from the incidence, we use the ONS population projections for England up to 2039.^12^ Average 5-yearly population estimates can be found in the appendix (p 9). Current cervical cancer rates were taken as an average of age-specific rates between 2011 and 2015,^5^ using the estimated mid-2014 population. Rates across ages were standardised with use of the European standard population.^13^

To validate the model, we applied weights so the model exactly replicated observed age-specific incidence of cervical cancer in England between 2011 and 2015.

We coded the microsimulation model in C++ (version 11), and we did the age period cohort model and data analyses using Stata (version 13.1).

### Scenarios

We explored four different policy scenarios using our model. The first scenario comprises no changes to current screening or vaccination coverage—ie, the so-called status quo scenario. In this scenario, age-specific screening coverage remains as currently observed up to 2040. Cytology screening is offered to women until 2019 when HPV screening is implemented. Implementation of HPV screening from 2019 means that a woman will first be offered this test between 2019 and 2023, depending on when she is next due to be screened. The second scenario considers the introduction of primary HPV screening. In this scenario, we compared cervical cancer incidences under the following scenarios: no HPV primary testing to assess effect of cytology only, the status quo (ie, primary HPV from 2019), bringing forward the introduction of HPV primary screening to 2017, and delaying the introduction until 2023. In the third scenario, unless otherwise stated, all assumptions remain same as the status quo scenario with the exception of the introduction of the nine-valent vaccine. Given that the government's contract for the supply of the four-valent vaccine ends in June, 2018, we have assumed that the earliest the nine-valent vaccine could be introduced to girls aged 12–13 years will be 2019.^14^ The last scenario involves changes to cervical screening coverage. Between 2011 and 2016, age-specific coverage decreased from 75·7% to 72·7% for those aged 25–64 years.^6^ Interventions to increase coverage have at best had a moderate effect (with the exception of offering vaginal self-sampling).^15^ Hence in addition to a scenario where screening coverage increases, we consider a decrease to show the importance of high population coverage. We hope that this decrease in coverage is not a realistic scenario, but one that it is important to understand the implications of.

When considering changes to screening coverage, it was increased or decreased from the current coverage at a steady rate to achieve the nominal coverage by 2031. The women who entered or exited the regularly screened group were equally split between the lapsed group and the never screened group. Details of how the coverage decreased in the scenario where screening is phased out completely can be found in the appendix (p 10). When screening coverage is increased, women who have never been screened become irregularly screened, and irregularly screened women become regularly screened. Similarly, when coverage is decreased, women who were regularly screened become irregularly screened, and irregularly screened women become never screened (ie, no screening in the past 15 years).

### Role of the funding source

This study was funded by Jo's Cervical Cancer Trust and the Cancer Research UK. The views expressed are those of the authors and not those of the funders. The funders of the study had no role in study design, data collection, data analysis, data interpretation, writing of the report, or decision to submit for publication. All authors had full access to the data. The corresponding author had full access to all the data in the study and had final responsibility for the decision to submit for publication.

## Results

**Figure 1:**
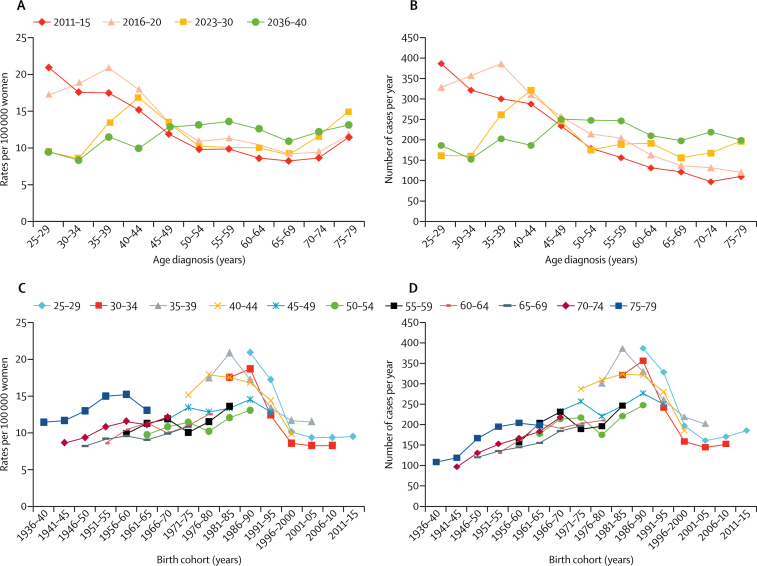
Rates of and yearly averages of cervical cancer under the status quo scenario (A) Cervical cancer rates per 100 000 women per age and year of diagnosis. (B) Yearly average numbers of cervical cancer cases per age and year of diagnosis. (C) Cervical cancer rates per 100 000 women per birth cohort and age of diagnosis. (D) Yearly average numbers of cervical cancer cases per birth cohort and age of diagnosis.

Figure 1 summarises the rates per 100 000 women per year and yearly average numbers of cervical cancer cases by year of diagnosis in the status quo scenario. Similar results by birth cohort are also illustrated in figure 1. The results from the status quo scenario show a shift in peak age of cancer diagnosis from 25–29 years in 2011–15 to 55–59 years (with a second peak at 75–79 years) in 2036–40. Unvaccinated women born between 1975 and 1990 were predicted to have a relatively high risk of cervical cancer throughout their lives. Consequently, cancers in these women will dominate cancer incidence over the next 30 years. By contrast, age-specific rates in women aged 30–34 years were reduced by 53% from 17·5 in 2011–15 to 8·3 per 100 000 women by 2036–40.

**Figure 2:**
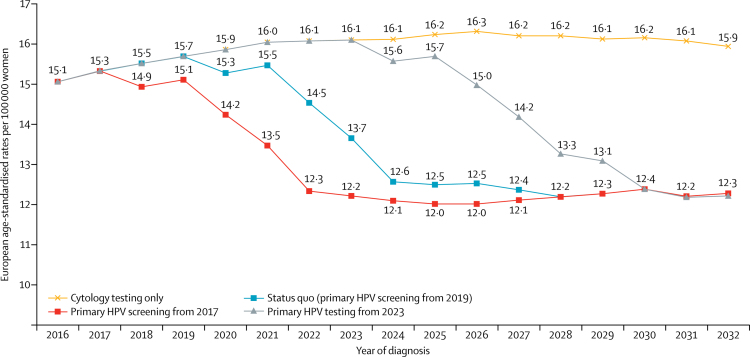
Effect of the introduction of HPV primary testing on age-standardised cervical cancer rates in women aged 25–64 years HPV=human papillomavirus.

Figure 2 shows the estimated reduction in European age-standardised rates per 100 000 women aged 25–64 years among the scenario for which HPV primary screening is introduced to the English cervical screening programme. Provided that HPV primary screening is introduced by 2023, we estimated that cervical cancer rates at ages 25–64 years could be reduced by 2·9 (19%) cases per 100 000 women by 2028–32 (from 15·1 in 2016 to 12·2) by introducing this new test. In absolute terms, delaying the introduction of HPV primary screening from 2017 to 2019 resulted in 1400 extra cervical cancers diagnosed in women aged 25–64 years between 2018 and 2028, because it takes 10 years for the full effect to be observed.

**Figure 3:**
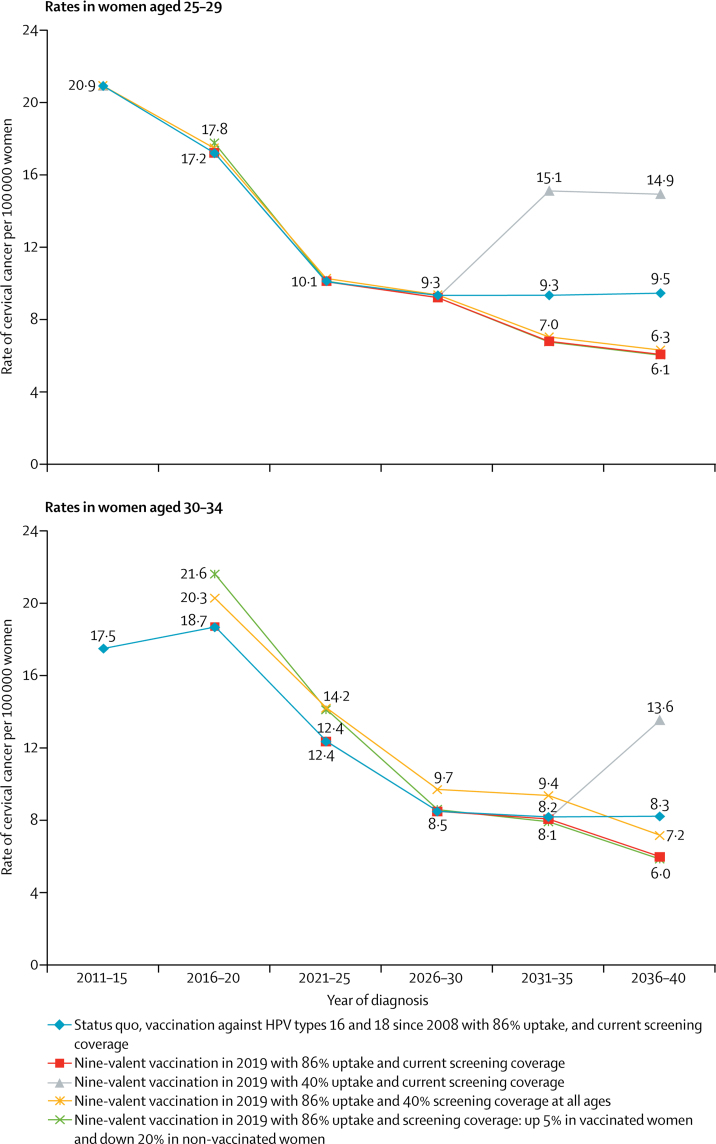
Effect of vaccine type, vaccine uptake, and screening coverage on cervical cancer rates Vaccination was introduced in England, UK, to girls aged 12–13 years in 2008–09 with a catch-up cohort aged 17–18 years (uptake in this cohort was poor). Not all women aged 30–34 years will have been vaccinated with the nine-valent vaccine by 2040, 24% will have received the four-valent vaccine instead.

Scenarios discussed from here onwards assume primary HPV screening is introduced in 2019, with all women (aged 25–64 years) offered screening by HPV testing by 2023. By 2021, all women aged 25–29 years will have been offered vaccination against HPV at age 12–13 years; therefore, changes to screening coverage make only a slight effect on cervical cancer rates in this age group. Cervical cancer rates per 100 000 women decreased by 55% from 20·9 in 2011–15 to 9·5 in 2036–40 among women aged 25–29 years vaccinated against HPV types 16 and 18 and by 71% from 20·9 in 2011–15 to 6·1 in 2036–40 among those receiving the nine-valent vaccine (figure 3). The additional benefit of the introduction of the nine-valent vaccine compared with continuing vaccination against HPV types 16 and 18 by 2036–40 was a further reduction of 36% in cervical cancer rates (ie, from 9·5 to 6·1 per 100 000 women in 2036–40). In this age group, the biggest modifier of cervical cancer risk in the future was vaccine uptake. In the most extreme scenario in which uptake of the nine-valent vaccine decreased to 40% by 2036–40, rates of cervical cancer were 144% higher (14·9 per 100 000 women) than the scenario in which uptake remained at 86% (6·1 per 100 000 women; figure 3).

Maintaining or increasing cervical screening coverage until 2025 in women aged 30–34 years will be important because few women will have been vaccinated before becoming sexually active. In fact, the effect of changes in coverage among this age group can be seen as early as 2016–20 (figure 3). The additional benefit of introducing the nine-valent vaccine compared with continuing HPV 16/18 vaccination by 2036–40 was a 28% reduction in cervical cancer rates (from 8·3 to 6·0 per 100 000 women). Among those vaccinated with the nine-valent vaccine cervical cancer rates decreased by 66% (from 17·5 to 6·0 per 100 000 women) and by 53% in those vaccinated against HPV types 16 and 18 (from 17·5 to 8·3 per 100 000 women) by 2036–40. If nine-valent vaccine uptake were to decrease to 40% in girls aged 12–13 years we would see its effect on cervical cancer incidences from 2036 onwards with a 127% increase in rates (from 6·0 when uptake is 86% to 13·6 per 100 000 women).

The current screening coverage and changes to regular screening of cervical cancer by age groups in England is summarised in table 1. Table 2 summarises the cumulative number of cancers and European age-standardised incidences in women aged 25–79 years. Because cervical screening is already preventing the majority of cervical cancers, the effect of screening was most apparent in the scenarios where the coverage decreased. Despite vaccination and primary HPV screening with current screening coverage, the European age-standardised rates of cervical cancer at ages 25–79 years showed only a moderate 10% decrease by 2036–40 (from 12·8 in 2011–15 to 11·6 per 100 000 women in 2036–40).

**Table 1:** Cervical cancer screening coverage by age group under various scenarios

	Current screening coverage*				**Absolute percentage coverage change (every 5 years) to achieve 85% regularly screened by 2031**		**Absolute percentage coverage change (every 5 years) to achieve 50% regularly screened by 2031**		**Absolute percentage coverage change (every 5 years) to achieve 20% regularly screened by 2031**	
	Regularly screened	Lapsed attender	Never screened	Total population	Regularly screened	Lapsed attender or never screened	Regularly screened	Lapsed attender or never screened	Regularly screened	Lapsed attender or never screened
25–29 years†	63·5%	··	36·5%	2 076 444	5·4%	−5·4%	−3·4%	3·4%	−10·9%	10·9%
30–34 years	70·4%	14·8%	14·8%	2 043 366	3·7%	−1·8%	−5·1%	2·6%	−12·7%	6·3%
35–39 years	73·1%	17·4%	9·5%	1 831 058	3·0%	−1·5%	−5·8%	2·9%	−13·3%	6·6%
40–44 years	75·1%	17·6%	7·3%	1 877 290	2·5%	−1·2%	−6·3%	3·1%	−13·8%	6·9%
45–49 years	75·2%	17·9%	6·9%	1 923 869	2·5%	−1·2%	−6·3%	3·2%	−13·8%	6·9%
50–54 years	80·8%	12·1%	7·1%	1 781 782	1·1%	−0·5%	−7·7%	3·9%	−15·2%	7·6%
55–59 years	74·6%	17·1%	8·3%	1 451 314	2·6%	−1·3%	−6·2%	3·1%	−13·7%	6·8%
60–64 years	72·4%	17·9%	9·7%	1 225 440	3·2%	−1·6%	−5·6%	2·8%	−13·1%	6·6%
65–69 years	72·4%	17·9%	9·7%	1 207 164	3·2%	−1·6%	−5·6%	2·8%	−13·1%	6·6%
70–74 years	72·4%	17·9%	9·7%	909 787	3·2%	−1·6%	−5·6%	2·8%	−13·1%	6·6%
75–79 years	72·4%	17·9%	9·7%	776 335	3·2%	−1·6%	−5·6%	2·8%	−13·1%	6·6%

* Observed in the 2014–15 cervical screening programme statistics;^6^ regularly screened defined as test within 3·5 years in those aged 25–49 years and within 5 years in those aged 50–64 years.

† Because women are first invited for screening at 25 years of age, women in this age group cannot be lapsed attenders.

**Table 2:** European age-standardised cumulative number of cancers and incidences per 100 000 women aged 25–79 years in various screening coverage scenarios and calendar years at diagnosis

	**2016–20**		**2021–25**		**2026–30**		**2031–35**		**2036–40**	
	N	Rate per 100 000	N	Rate per 100 000	N	Rate per 100 000	N	Rate per 100 000	N	Rate per 100 000
Currently observed (status quo)	947	14·0	863	12·8	785	11·6	794	11·8	782	11·6
85% coverage	906	13·4	786	11·7	685	10·1	663	9·8	653	9·7
50% coverage	1043	15·5	1049	15·5	1034	15·3	1131	16·8	1112	16·5
20% coverage	1161	17·2	1273	18·9	1333	19·8	1532	22·7	1506	22·3
Phasing out*	2463	36·5	2498	37·0	2363	35·0	2527	37·4	2558	37·9

* In this scenario, screening is no longer offered from 2016 onwards; it takes several years for women who had been regularly screened to eventually become never screened because they must become lapsed attenders first. Hence, some degree of protection against cervical cancer remains in this population until 2031–35 (appendix p 10).

## Discussion

As a public health policy, HPV immunisation will deliver the biggest reduction in cervical cancer diagnosed. Provided vaccine uptake is maintained, and even without the introduction of the nine-valent vaccination, cervical cancer rates in women aged 25–34 years will decrease by more than 50%. Introduction of the nine-valent vaccine from 2019 would decrease cancer rates by a further 36% in women aged 25–29 years and 28% in those aged 30–34 years by 2036–40. However, in the next 25 years, the vaccination strategy will have no direct effect on women born before 1991 who were not vaccinated before HPV exposure. In the short term, the timeliness of the introduction of HPV primary screening into the screening programme will be the most important determinant of the potential reduction in the number of cervical cancers diagnosed among unvaccinated women. Unfortunately, the risk of acquiring an HPV infection that will progress to cancer has increased in unvaccinated individuals born since 1960 (data sourced from the Genitourinary Medicine Clinic Activity dataset and Public Health England [KC60]), suggesting that current screening coverage is not sufficient to maintain—much less reduce—cervical cancer incidence in the next 20 years.

Age period cohort models have been used to estimate the impact of screening by assuming that observed period effects reflect the outcome of screening.^16^, ^17^ Dynamic transmission models^18^ consider the effects of HPV prevalence and transmission, cervical screening, and HPV vaccination on cervical cancer incidence. For absolute efficacy, they need to be carefully calibrated to each population but relative efficacy is largely invariant to calibration. The current study combined an age cohort model with a dynamic model to estimate the effect of screening, taking into account screening attendance and vaccination coverage. We used population-based data to estimate the risk of being diagnosed with cervical cancer by screening attendance. This method enabled the estimation of absolute changes to cervical cancer incidence and allowed us to vary screening coverage and vaccine uptake to estimate absolute effect on rates. We know of no similar studies with cancer as an outcome, although we did identify a similar study with stroke as the main outcome.^19^ Other studies of cervical cancer in the UK use just one type of modelling; these include an economic evaluation of HPV vaccination,^20^ a dynamic model exploring the effect of HPV vaccination,^21^ and a microsimulation study considering screening in England following the introduction of nine-valent vaccination.^22^ None of them have estimated age-specific absolute risks by calendar time.

The age cohort model used to underpin this research was adapted to ensure that cervical cancer rates in the absence of screening were not predicted to increase substantially beyond the greatest rates observed historically. This constraint was particularly important for more recent cohorts, which have seen rates in young women more than double in the past 15 years. We have assumed, on the basis of published literature,^23^, ^24^ that opportunistic screening had no effect on cervical cancer incidence before 1988. However, if screening before 1988 did have an effect, our age period cohort model will have underestimated cervical cancer rates in the absence of screening up to 2040. This effect would mostly affect birth cohorts born before 1955.

It was recently estimated, using individual level screening history data, that in the absence of screening, cervical cancer incidences in England would be 2·5 times higher than current rates.^25^ The projected increases in incidence from the age cohort model used in our study suggest a similar (3·1-times) increase in rates (appendix pp 2, 3). Although the benefit of screening in the model could be overestimated because of a higher underlying risk of cervical cancer in women who do not attend screening, given the similarity between the age cohort estimates and those from individual level screening data, the magnitude of the overestimation must be small.

The model does not take into account the effect of herd immunity. Because of sexual mixing patterns, the majority of the impact of herd immunity would occur in women born after 1985, so its effect on overall rates will be small. It will be more substantial when looking at age-specific rates, particularly where vaccine uptake decreases. We also do not account for type replacement (eg, from HPV types 16 and 18 to HPV 59), which would diminish the effect of HPV 16/18 vaccination.^26^ Furthermore, the model does not take into account changes to the effectiveness of cervical screening by cytology. On the one hand, with the prevalence of disease decreasing among vaccinated women, and less cytology taking place following the introduction of HPV screening, it is possible that the cytology workforce might become less skilled, leading to a decreased effect of screening. On the other hand, fewer cytoscreeners looking only at HPV-positive samples (in which high-grade precancerous lesions will be more common) might increase the sensitivity of cytology.

The multidisciplinary nature of the screening programme means that a great number of organisations and individuals are involved in delivering the programme, which has resulted in extensive piloting and planning so that there has been wide variation in when different programmes have introduced HPV testing in primary cervical screening. For example, Kaiser Premanente Northern California introduced primary HPV screening in combination with primary cytology in 2003.^27^ The Netherlands, Sweden, and Australia are expected to roll out HPV primary screening by the end of 2017.^28^, ^29^ In England, six pilot sites covering about 5% of the population have been offering primary HPV screening since 2013. In 2015, baseline results from the pilot sites confirmed that screening by HPV primary screening achieved a higher detection rate of CIN 2 (cervical squamous intraepithelial neoplasia 2) or worse than does cytology screening.^30^ National roll-out is planned for 2019.^31^ The complexity of implementing any new intervention into a successful screening programme and the potential for harm has in the past led to a cautious reaction to change. For example, the roll-out of liquid-based cytology took almost 5 years.^32^ In our model, we estimated that failure to introduce HPV testing nationally in 2017 will lead to the missed opportunity of preventing about 1400 cervical cancer cases between 2018 and 2028.

Increasing screening coverage in unvaccinated women will remain a considerable challenge for the programme. We have assumed that increasing coverage brings in never-screened women and lapsed attenders in equal proportions. Engagement of women who have never attended screening is substantially harder than convincing women who have previously engaged with the programme.^33^, ^34^ There is concern that cervical cancer will become a disease of the underprivileged. Marginalised women are less likely to be vaccinated and have lower awareness of screening than those who are not marginalised.^35^ Engagement with these women is essential.

Once HPV primary screening is fully introduced, screening intervals are expected to increase to at least 5-yearly at all ages.^36^ The cost-effectiveness of delivering HPV screening once every 5 years to vaccinated women needs to be reassessed because there is evidence to suggest that one test every 10 years might be sufficient.^37^ Furthermore, it seems possible that the programme will use new technologies, such as genotyping or DNA methylation testing, which will allow for individual risk-profiling with variable time to next screening invitation depending on risk. Vaccination of women older than 25 years has been proposed; however, the protection offered to these women appears to be considerably lower than the protection observed in women aged 12–13 years.^38^ Clear screening campaign messages will become essential if we are to continue engaging women with screening.

To meet these challenges, policy makers need to ensure that there is a well thought-out mechanism to introduce change into the screening programme swiftly and effectively without compromising quality.
