# Supplementary material for: Prediction of cervical cancer incidence in England, UK, up to 2040, under four scenarios: a modelling study
Source: Lancet Public Health. 2017 Dec 19;3(1):e34–43. doi: 10.1016/S2468-2667(17)30222-0 (PMC5765529; doi:10.1016/S2468-2667(17)30222-0)
Supplement: Supplementary appendix [file mmc1.pdf]

THE LANCET
Public Health

Supplementary appendix

This appendix formed part of the original submission and has been peer reviewed. We post it as supplied by the authors.

Supplement to: Castanon A, Landy R, Pesola F, et al. Prediction of cervical cancer incidence in England, UK, up to 2040, under four scenarios: a modelling study. *Lancet Public Health* 2017; published online Dec 18. http://dx.doi.org/10.1016/S2468-2667(17)30222-0.

Appendix

Summary of the main components of the model

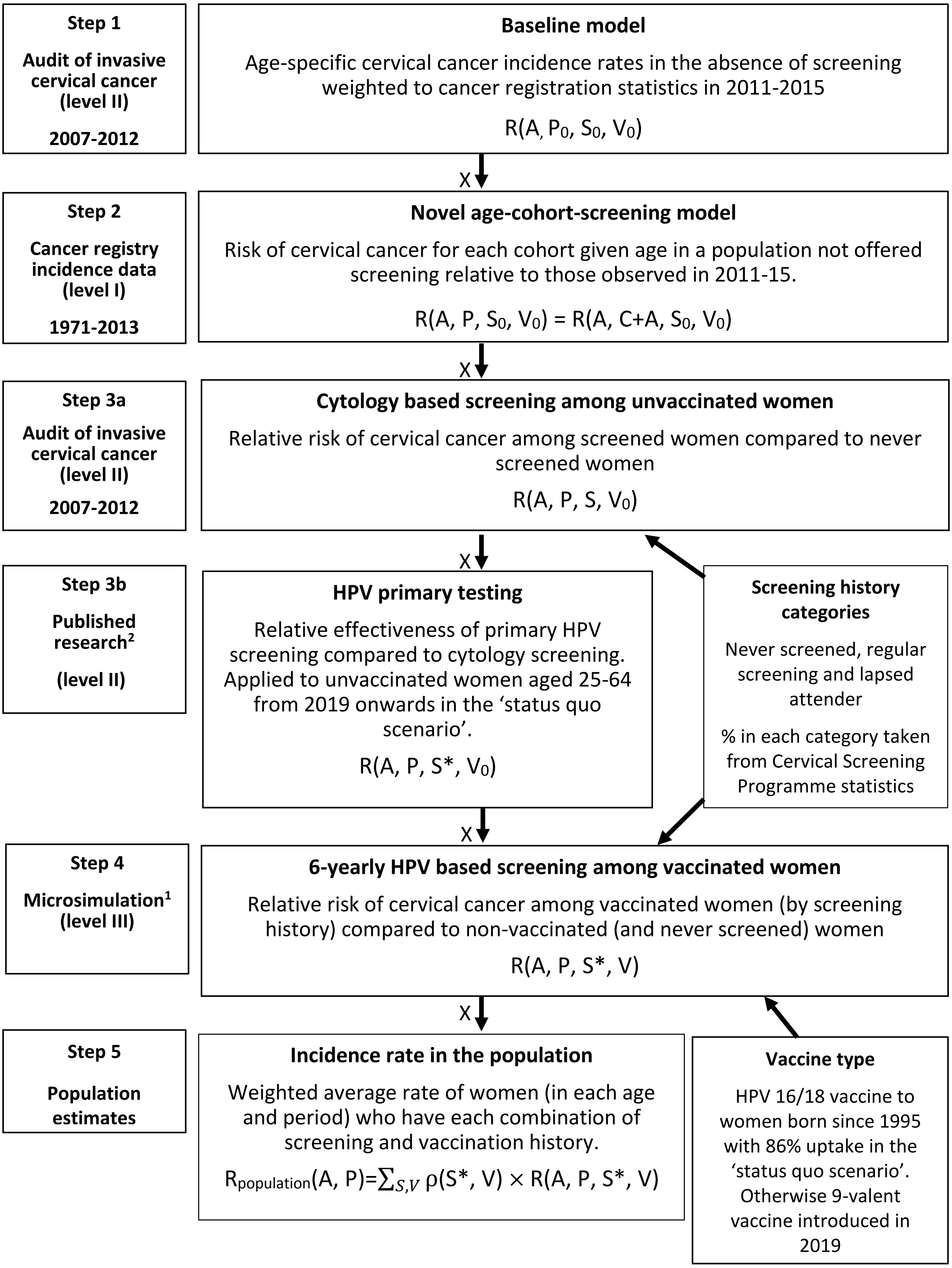

Figure 1. Legend: Step by step summary of how the different components of the model were brought together. Level I refers to modelling using population cancer incidence data. Level II using individual woman data on screening exposure and cervical cancer diagnosis. Level III uses microsimulation to study the theoretical effect of screening and vaccination based on underlying natural history. In the notation R is the risk, A is age, C is cohort, P is period, P_0 is baseline period 2007-12, ρ is proportion, S_0 is no screening, S^* allows either cytology or HPV screening, V_0 is no vaccination.

Age-period-cohort model. Predicted cervical cancer incidence rates up to 2040 assuming no effect of screening

We have fitted a novel age-period-cohort (APC) model to cervical cancer incidence from 1971 to 2013, and extrapolated over the next 25 years assuming a Poisson distribution of cancer events. Data on 125,348 women diagnosed with cervical cancer age 25 to 79 from routinely collected statistics on cervical cancer (ICD-10 C53) from 1971 to 2013 were included.[3] The APC approach is based on a log-linear model for the expected cancer rates with additive components representing the temporal effects of age, period and cohort.[4] The idea of APC models is to approximate the rate, which is a bivariate function of age and period, by an additive combination of univariate functions of age, period and cohort.[5]

$$\mathrm{g}\big(\gamma(age, period)\big) = f_A(\mathrm{age}) + f_P(\mathrm{period}) + \ f_C(\mathrm{cohort}) \qquad (1)$$

where γ is the incidence rate, f_A, f_P and f_C are functions of age, period (i.e year of incidence) and cohort (i.e year of birth) and g in the link function. We used a log link which sets the rate to be exponential. With a log-link, we have:

$$\mathrm{incidence} = H \times \exp\{f_P(\mathrm{period})\} \times \ \exp\{f_C(\mathrm{cohort})\} \qquad (2)$$

The functions f_A, f_P and f_C were set to be natural cubic splines as they allow us to model more realistic trends and projections under the assumption that changes occur smoothly rather than in sudden jumps.

Cohort effects reflect changes in risk factors across different birth cohorts. If $\exp\{f_C(1950) - f_C(1930)\}$=2, this implies that at all ages those born in 1950 have twice the risk of those born in 1930. For cervical cancer, cohort effects reflect changes in sexual practices increasing the likelihood of infection by HPV virus which is known to be a necessary cause of cervical cancer. To capture the fact that not only a higher proportion of women are exposed to HPV, but that women are becoming exposed to HPV at younger ages, we have also applied a horizontal shift to the cohort effect, which correspond to age of first exposure. We applied a 5-week shift per year of birth to take into account earlier exposure to the HPV virus. The shift is applied to women born after 1955 and we cap the shift at a maximum of 5 years. We use the following formula:

Model age = true age + (cohort-1955)*5/52 for cohorts born after 1955

Model age = true age for earlier cohorts

We then substitute “model age” for “age” in (1). The model is set up to stop splines from changing beyond 2013. This was done since it is the last year of observed data and the long-term effect of vaccination on cervical cancer rates are not known. Similarly, geometric damping was applied to birth cohorts after 1981.

The period effect was set to 0 prior to the introduction of screening in 1988. To estimate incidence rates in the absence of screening, we fit the full APC model (1) including f_A, f_P and f_C, but then estimated counterfactual rates using fitted f_A and f_C only. Figure S1 below compares observed rates to projections based on the modified APC model described above with a 5-week HPV-age adjustment.

Figure S1. Observed European age standardised cervical cancer rates per 100,000 women from 1970 to 2013 in England at ages 20 to 79 and projected rates in the absence of screening up to 2040.

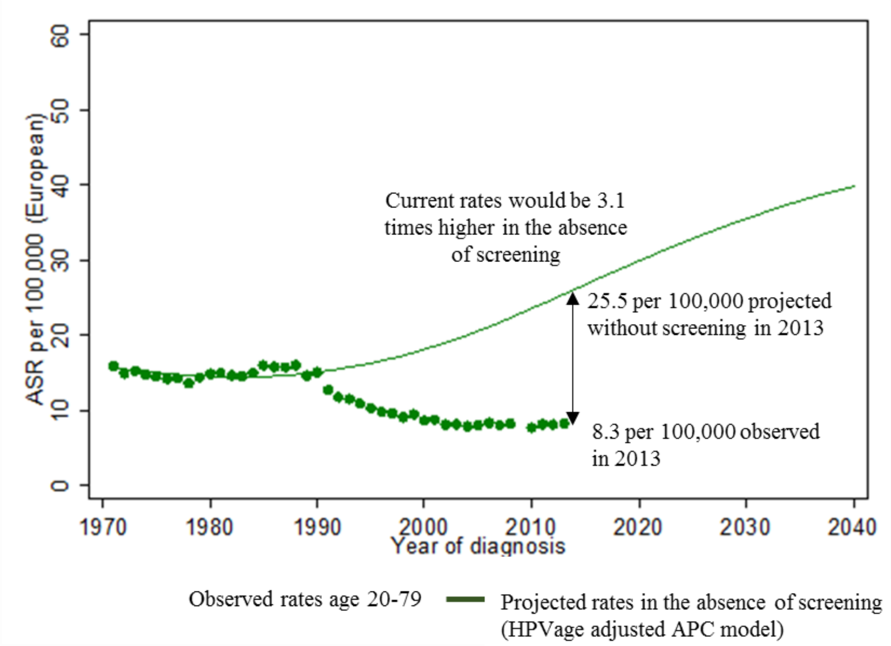

Relative effectiveness of primary HPV screening compared to cytology screening.

We assumed a sensitivity of HPV testing in cytology negative women of 80%, and 97% in cytology positive women, giving an overall sensitivity of HPV testing of 93%. Further we assume that HPV testing does not prevent any additional cancers within 18 months of the test (these would have been diagnosed symptomatically or through cytology screening). For each screening history category we estimate (from the audit) the proportion (P) of cancers with a negative cytology 18 months to 6 years before diagnosis and apply the sensitivity (S) of HPV testing (in cytology negative women) to this to estimate the additional impact of HPV testing.[6] The rate (relative to the rate with conventional screening) is then (1-P)+P(1-S).

Technical report on the microsimulation model used in this manuscript

Unusually we use a model that combines three levels of modelling: 1) Population level, we use incidence trends to run an age cohort model to ascertain what rates would be like in the absence of screening; 2) Individual level observable data, we use a case-control study of cervical cancer screening histories; and 3) Unobservable individual level data, we model this using a microsimulation.

Note that calibration is mostly only an issue for the microsimulation part of the model and we (usually) use a combination of all three components of the model in this manuscript.

Natural history

We have developed a microsimulation model[1] to simulate the natural history with respect to HPV and cervical cancer for 1 million women, which begins at age 12 when all women are assumed to be HPV negative. Women can transition between states every 6 months until age 80. We do not allow for hysterectomies or deaths prior to age 80, nor for new HPV infections beyond age 65. The possible states are shown in Figure S2 below, and are 'Susceptible' (HPV negative), 'new (HPV', 'persistent HPV', 'low-grade cervical intraepithelial neoplasia (CIN)', 'high-grade CIN', 'asymptomatic cancer' and 'symptomatic cancer'. We use 'asymptomatic' to refer to cancers that are only diagnosed as a result of screening, and 'symptomatic' cancers are diagnosed without (or despite) screening, at the time of transition into the state of 'symptomatic cancer'. In line with scientific evidence [7, 8], we assume that cervical cancer cannot occur without HPV infection.

We have divided HPV strains into 16/18 and other high risk HPV. As well as progressing to more advanced states, it is also possible to regress to less advanced states: from 'HPV positive' to 'HPV negative', 'low grade CIN' to 'HPV negative', and 'high grade CIN' to 'HPV negative'. The transition probabilities are HPV-type and age dependent. The model is not completely Markov, as the probability of regressing or progressing from an HPV-positive state depends on the time since HPV infection. We used an iterative process to estimate the transition probabilities, using estimates in the literature as a starting point [9], until the simulated HPV prevalence rates were within 15% (relative) of the published age-specific HPV prevalence data from the ARTISTIC trial in England [10] (Figure S3). We also ensured the proportion of cancers caused by high-risk HPV[11] and the lifetime risk of cervical cancer in the absence of screening produced realistic results for England – as the lifetime risk in the absence of screening is unobservable, we used data from Finland in 1972-6 as the lower limit and Brazil in 1973 as the upper limit [12]. The six-monthly transition probabilities are given in Table S5 below.

The model was run independently for HPV16/18 and non-16/18 HPV types, and the results were combined, by taking the more advanced state at each time point. This allowed us to calibrate the simulated data to observed overall HPV prevalence. We assumed that 10% of the population had a higher risk of becoming infected with both HPV-16/18 and non-16/18 HPV strains; this was modelled by increasing the 'HPV negative' to 'HPV positive' probability for both HPV16/18 and other high risk HPV infections by 20% for these individuals.

Probabilities and random numbers were generated using a Mersenne Twister, and the model was implemented in C++.

HPV Vaccination

HPV vaccination was introduced in England in 2008. Initially girls aged 12/13 were offered the vaccine in school, though a 'catch up' cohort of women aged 14-18 were also offered the vaccine. In all vaccination cases,

we assume all three doses of the vaccine occurred at age 12, prior to infection with HPV. We use results from three vaccination scenarios:

1. No vaccination
2. The vaccine currently used in England (Gardasil), is a quadrivalent vaccine which we assume in the simulation initially prevents all HPV 16/18 (as well as HPV 6/11, which are associated with genital warts, but not cervical cancer), but wanes by 0.25% (absolute) every year, and initially prevents 15% of other high risk HPV strains (cross-protection), waning by 0.0375% per year. We estimated the level of cross-protection for the quadrivalent vaccine from a weighted average of the level of protection against 6 month type-specific HPV persistence [13], weighted by HPV type prevalence in cancers not caused by HPV16/18 [14].
3. The vaccine which is very likely to be introduced in England in the coming years, a nonavalent vaccine Gardasil-9, preventing all HPV 16/18 initially, but waning by 0.25% every year, and initially preventing 67% of other high risk HPV strains, waning by 0.1675% per year.

Screening programme and treatment

We consider two levels of screening (regular screening and lapsed screening), as well as cancer risk in the absence of screening. In regular screening, everyone attends when they are invited. In lapsed screening, women attend one or two out of every three consecutive rounds of screening they are invited to. Initially unvaccinated women are invited for screening by cytology every 3 years if aged 25-49 and 5-yearly if aged 50-64, though we consider the effect of implementing HPV testing at a range of years. Vaccinated women are invited for 6-yearly HPV testing with cytology triage (i.e. a cytology test if the HPV test is positive) from ages 25-64.

At each routine screening event an HPV test with 96% sensitivity was performed[15]; for those who tested HPV positive a cytology test was carried out, with 70% sensitivity for low-grade CIN, 85% for high-grade CIN (the sensitivity to CIN2+ was 85.6% in a trial[16] and 92% for asymptomatic cancer.

If the cytology test was positive, then women were assumed to be treated successfully in 90% of high-grade CIN cases (allowing for some treatment failure and some women not to return for treatment). 5% of low-grade CIN was also assumed to be treated successfully. Women who were HPV positive but cytology negative were recalled one year later, and if still HPV positive recalled once more one year later. After three consecutive HPV-positive and cytology-negative results, women were referred to colposcopy, where 90% of high-grade CIN was treated successfully, and women with HPV or low-grade CIN were invited back a year later.

Once successfully treated, women who would have developed cancer in the absence of screening had a 0.09% per annum chance of developing cancer [17].

Cancer is diagnosed when transition occurs into the symptomatic cancer state, or when cancer is detected through screening. Therefore in the absence of screening only symptomatic cancers are diagnosed.

Figure S2: Natural history states and possible transitions in the microsimulation model

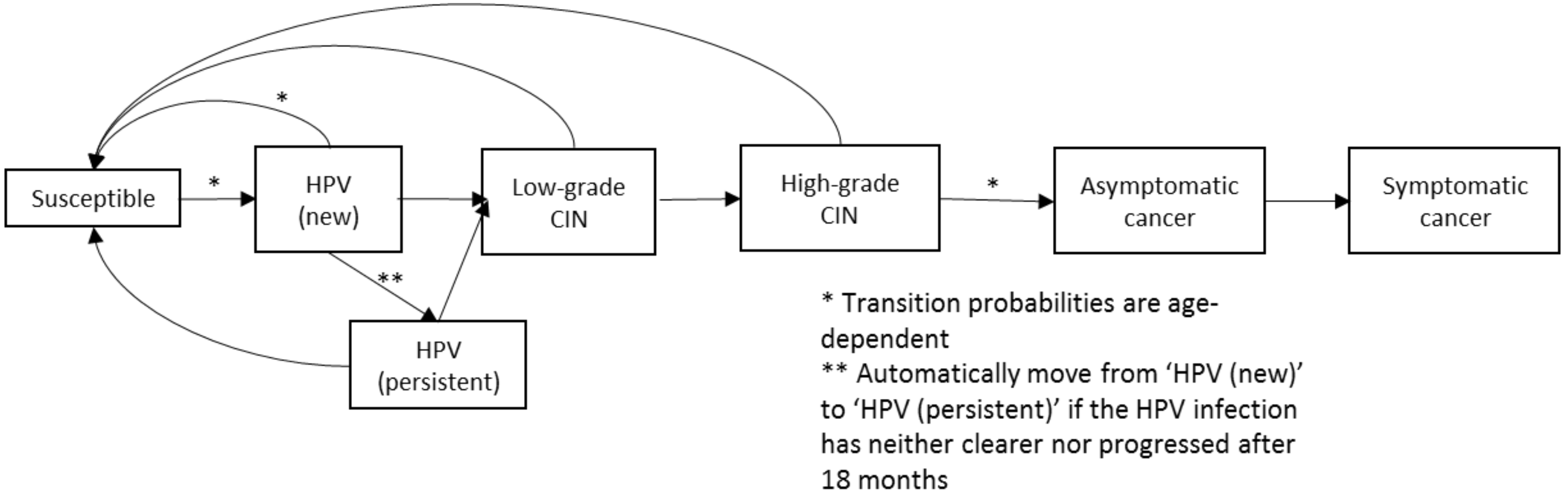

Figure S3: Validation of simulated HPV prevalence to observed HPV prevalence in the ARTISTIC study[10], by age

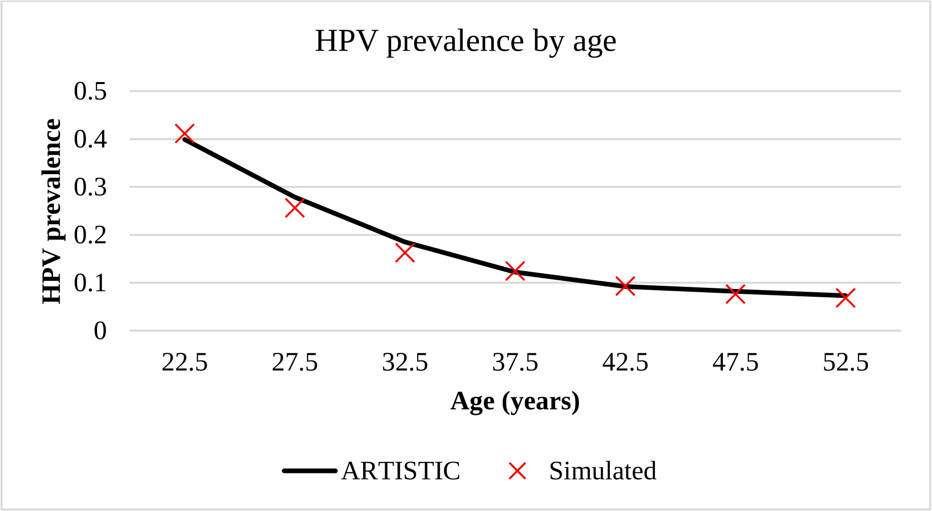

Six-monthly transition probabilities between natural history states

**Transition**	**High risk HPV (16/18)**	**All other high risk HPV**
	Probability	Probability
Susceptible to HPV positive		
Under 18 years old	0.035	0.031
18 – 23	0.049	0.045
24 – 29	0.016	0.014
30 – 39	0.0055	0.004
40 – 54	0.0025	0.0018
55 – 64	0.0012	0.0012
HPV positive to low-grade CIN	0.062	0.04
New (< 18 months) HPV □ Susceptible		
Under 23 years old	0.21	0.22
23 – 34	0.27	0.28
35 +	0.27	0.28
Persistent HPV to Susceptible	0.119	0.119
Low-grade CIN to Susceptible	0.172	0.182
Low-grade CIN to high-grade CIN	0.03	0.03
High-grade CIN to Susceptible	0.01	0.01
High-grade CIN to Asymptomatic cancer		
Under 30 years old	0.0015	0.0015
30 – 34	0.0025	0.0025
35 – 39	0.0035	0.0035
40 – 49	0.0065	0.0065
50 – 61	0.009	0.009
62 +	0.011	0.011
Asymptomatic cancer - symptomatic cancer	0.145	0.145

Introduction of primary HPV screening into the cervical screening programme

	Primary HPV screening rolled out in all laboratories in England in year 1									
Age	Year 1 (HPV introduced)	Year 2*	Year 3*	Year 4Ϯ	Year 5	Year 6±	Year 7	Year 8	Year 9	Year10
25-49	0%	13%	13%	13% (87%x1/3[£])	13% (87%x2/3[£])	13% (87%x3/3[£])				
50-64	0%	10%	10%	10%	10%	10% (90%x1/5)	10% (90%x2/5)	10% (90%x3/5)	10% (90%x4/5)	10% (90%x5/5)

*HPV will have an effect among those asked to return earlier due to a positive HPV test/negative cytology. The proportion of women aged 25-49 on cytological surveillance is 13% and those age 50-64 is 10%.[18]

Ϯ Reduction in cervical cancer age 25-29 first observed

± Reduction in cervical cancer age 50-64 first observed

[£] In any given year 33% of women under age 50 will be invited for screening (20% of those age 50-64). Hence in year 4, 87% of the 33% (because 13% were already on early recall) will attend for screening (having been first screened in year1).

Vaccine coverage, birth cohort, proportion protected and year they enter the screening programme.

Birth cohort	Coverage[1]	Vaccine age	% protected[3]	Year enter screening
01 Sept 95 – 31 Aug 97	0·86[2]	12/13	100	2020-2022
01 Sept 94 – 31 Aug 95	0·685	14/15	95	2019
01 Sept 93 – 31 Aug 94	0·686	15/16	90	2018
01 Sept 92 – 31 Aug 93	0·417	16/17	85	2017
01 Sept 91 – 31 Aug 92	0·389	17/18	80	2016
01 Sept 90 – 31 Aug 91	0·474	17/18	80	2015

[1]Coverage for the catch-up cohort taken from national statistics[19].

[2]Three dose HPV coverage among women vaccinated age 12/13 [20].

[3]Based on data from Mesher et al[21] which shows that the prevalence of HPV16/18 in women aged 16 to 18 is 19·1 and it remains around the 17-20% mark up to age 25.

Table S3. Average 5 year population estimates used to calculate cervical cancer rates[22]

	2014	2016-20	2021-25	2026-30	2031-35	2036-40
25 to 29	1857200	1905420	1946340	1715900	1819560	1956025
30 to 34	1862900	1902560	1946340	1860480	1753600	1837225
35 to 39	1701200	1847400	1908860	1950740	1865380	1761925
40 to 44	1869000	1728300	1845540	1905820	1947880	1873800
45 to 49	1983100	1901580	1725060	1841540	190210	1947925
50 to 54	1877400	1968580	1891740	1717800	1834440	1889825
55 to 59	1610400	1806560	1947240	1873080	1703380	1811150
60 to 64	1487800	1552020	1773600	1913620	1843520	1676200
65 to 69	1528900	1476600	1506980	1725000	1864300	1815925
70 to 74	1146800	1388640	1404700	1439120	1651920	1791475
75 to 79	964600	1017460	1275980	1298120	1337980	1520150
All ages	17889300	18495120	19172380	19241220	17812170	19881625

Table S4. Coverage by age group in the scenario where screening is phased out from the population.

	2016-20			2021-25			2026-30			2031-35			2036-40		
	Never	Lapsed	Regular	Never	Lapsed	Regular	Never	Lapsed	Regular	Never	Lapsed	Regular	Never	Lapsed	Regular
25-29	0·64	0·06	0·30	1·00	0·00	0·00	1·00	0·00	0·00	1·00	0·00	0·00	1·00	0·00	0·00
30-34	0·72	0·16	0·12	0·64	0·35	0·01	1·00	0·00	0·00	1·00	0·00	0·00	1·00	0·00	0·00
35-39	0·75	0·18	0·08	0·78	0·21	0·00	0·64	0·37	0·00	1·00	0·00	0·00	1·00	0·00	0·00
40-44	0·77	0·17	0·06	0·82	0·18	0·00	0·84	0·16	0·00	0·91	0·09	0·00	1·00	0·00	0·00
45-49	0·77	0·18	0·06	0·84	0·16	0·00	0·89	0·11	0·00	0·96	0·04	0·00	1·00	0·00	0·00
50-54	0·82	0·12	0·06	0·85	0·14	0·01	0·93	0·07	0·00	0·98	0·02	0·00	1·00	0·00	0·00
55-59	0·76	0·17	0·07	0·87	0·12	0·01	0·93	0·07	0·00	0·99	0·01	0·00	1·00	0·00	0·00
60-64	0·74	0·18	0·09	0·84	0·15	0·01	0·93	0·07	0·00	0·99	0·01	0·00	1·00	0·00	0·00
65-69	0·74	0·18	0·09	0·84	0·15	0·01	0·93	0·07	0·00	0·99	0·01	0·00	1·00	0·00	0·00
70-74	0·74	0·18	0·09	0·84	0·15	0·01	0·93	0·07	0·00	0·99	0·01	0·00	1·00	0·00	0·00
75-79	0·74	0·18	0·09	0·84	0·15	0·01	0·93	0·07	0·00	0·99	0·01	0·00	1·00	0·00	0·00

References

1. Landy R, Windridge P, Gillman MS, Sasieni PD. What cervical screening is appropriate for women who have been vaccinated against high risk HPV? A simulation study. *Int J Cancer*. 2017.
2. Castanon A, Landy R, Sasieni P. By how much could screening by primary human papillomavirus testing reduce cervical cancer incidence in England? *J Med Screen*. 2017;24(2):110-2.
3. Cancer Registration and Analysis Service. Cervical cancer incidence in single year of age. In: Obtained by special request (ODR_2014_335) from Public Health England (PHE), editor. 2016.
4. Holford TR. The estimation of age, period and cohort effects for vital rates. *Biometrics*. 1983;39(2):311-24.
5. Sasieni P. Age-period-cohort models in Stata. *Stata Journal,*. 2012;12(1):45-60.
6. Castanon A, Landy R, Sasieni P. By how much could screening by primary human papillomavirus testing reduce cervical cancer incidence in England? . *J Med Screen*. 2016.
7. Franco EL, Rohan TE, Villa LL. Epidemiologic evidence and human papillomavirus infection as a necessary cause of cervical cancer. *JNCI-J Natl Cancer I*. 1999;91(6):506-11.
8. Bosch F, Lorincz A, Munoz N, Meijer C, Shah K. The causal relation between human papillomavirus and cervical cancer. *J Clin Pathol*. 2002;55(4):244-65.
9. Goldie SJ, Grima D, Kohli M, Wright TC, Weinstein M, Franco E. A comprehensive natural history model of HPV infection and cervical cancer to estimate the clinical impact of a prophylactic HPV-16/18 vaccine. *Int J cancer*. 2003;106(6):896-904.
10. Kitchener H, Almonte M, Gilham C, et al. ARTISTIC: a randomised trial of human papillomavirus (HPV) testing in primary cervical screening 2009 [33]. Last accessed:
11. Smith JS, Lindsay L, Hoots B, et al. Human papillomavirus type distribution in invasive cervical cancer and high-grade cervical lesions: A meta-analysis update. *Int J Cancer*. 2007;121(3):621-32.
12. International Agency for Research on Cancer. Cancer Incidence in Five Continents, Volume IV. Waterhouse J; Muir C; Shanmugaratnam K; Powell J, eds. Lyon 1982.
13. Brown DR, Kjaer SK, Sigurdsson K, et al. The impact of quadrivalent human papillomavirus (HPV; types 6, 11, 16, and 18) L1 virus-like particle vaccine on infection and disease due to oncogenic nonvaccine HPV types in generally HPV-naive women aged 16–26 years. *J Infect Dis*. 2009;199(7):926-35.
14. Clifford G, Franceschi S, Diaz M, Munoz N, Villa LL. HPV type-distribution in women with and without cervical neoplastic diseases. *Vaccine*. 2006;24:S26-S34.
15. Arbyn M, Ronco G, Anttila A, et al. Evidence regarding human papillomavirus testing in secondary prevention of cervical cancer. *Vaccine*. 2012;30 Suppl 5:F88-99.
16. Bergeron C, Giorgi-Rossi P, Cas F, et al. Informed cytology for triaging HPV-positive women: substudy nested in the NTCC randomized controlled trial. *J Natl Cancer Inst*. 2015;107(2).
17. Soutter W, de Barros Lopes A, Fletcher A, et al. Invasive cervical cancer after conservative therapy for cervical intraepithelial neoplasia. *The Lancet*. 1997;349(9057):978-80.
18. Health and Social Care Information Centre. Cervical Screening Programme, England - 2013-14. 2014 [updated November 25, 2014. Available from: http://www.hscic.gov.uk/catalogue/PUB15968. Last accessed: 01 July 2016.
19. Sheridan A, White J. Annual HPV vaccine coverage in England in 2009/2010. 2010 [Available from: https://www.gov.uk/government/publications/annual-hpv-vaccine-coverage-in-england-in-2009-2010. Last accessed:
20. Public Health England (PHE). Human Papillomavirus (HPV) Vaccine Coverage in England, 2008/09 to 2013/14. A review of the full six years of the three-dose schedule. : Immunisation, Hepatitis and Blood Safety Department.; 2015 [updated 12 July 2017. Available from: https://www.gov.uk/government/publications/human-papillomavirus-hpv-immunisation-programme-review-2008-to-2014. Last accessed: 12 July 2017

21. Mesher D, Cuschieri K, Hibbitts S, et al. Type-specific HPV prevalence in invasive cervical cancer in the UK prior to national HPV immunisation programme: baseline for monitoring the effects of immunisation. *J Clin Pathol*. 2015;68(2):135-40.
22. Nash A. Subnational Population Projections for Regions in England: 2014-based population projections, Table 1: England and Regions, 5 year age groups, Females. : Office for National Statistics,; 2016 [Last accessed: 27 march 2017.
